# The Relationship Between Cardiac Syndrome X and Obstructive Sleep Apnea and the Effects of Sleep Apnea Treatment on Myocardial Ischemia

**DOI:** 10.3390/jcm14165897

**Published:** 2025-08-21

**Authors:** Umit Ozturk, Beste Ozben, Mustafa Kursat Tigen, Baran Balcan, Tunc Ones, Gulin Sunter, Nuh Filizoglu, Murat Sunbul, Emre Gurel, Altug Cincin

**Affiliations:** 1 Department of Cardiology, Marmara University, 34899 Istanbul, Turkey; besteozben@yahoo.com (B.O.); mktigen@yahoo.com (M.K.T.); drsunbul@yahoo.com.tr (M.S.); emregurelctf@yahoo.com (E.G.); acincin@yahoo.com (A.C.); 2 Faculty of Medicine, Department of Pulmonology, Koç University, 34450 Istanbul, Turkey; drbaranbalcan@gmail.com; 3 Department of Nuclear Medicine, Marmara University, 34899 Istanbul, Turkey; tones@marmara.edu.tr; 4 Department of Neurology, Marmara University, 34899 Istanbul, Turkey; ygulin@yahoo.com; 5 Department of Nuclear Medicine, Istanbul Lütfi Kırdar Training and Research Hospital, 34760 Istanbul, Turkey; nuhfilizoglu@gmail.com

**Keywords:** cardiac syndrome X, sleep apnea, microvascular angina, angina, continuous positive airway pressure, ischemia

## Abstract

**Background/Objectives:** Cardiac Syndrome X (CSX) is associated with significant physical and psychiatric morbidity despite no obvious effect on long-term mortality. Obstructive sleep apnea (OSA) is a prevalent condition in close association with numerous cardiovascular diseases. The precise relation between CSX and OSA remains unclear. The aim of this study is to explore the relation between OSA and CSX, as well as the impact of continuous positive airway pressure (CPAP) therapy on myocardial ischemia. **Methods:** This single-center prospective cohort study examined patients who were selected consecutively from the Cardiology Outpatient Clinic with angina or angina-equivalent complaints and with ischemia on myocardial perfusion scintigraphy (MPS), and who were subsequently diagnosed with CSX via coronary angiography. Patients with previous myocardial infarction and previous percutaneous coronary intervention or coronary artery by-pass grafting surgery were excluded, since these conditions could not be regarded as CSX. The presence of OSA was explored by polysomnography (PSG). CPAP therapy was applied for three months to those diagnosed with OSA. Following a three-month course of treatment, a myocardial perfusion scintigraphy (MPS) was conducted, to assess myocardial ischemia. The IBM^®^ SPSS Statistics Version 26 software was employed for the purpose of statistical analysis. **Results:** Among the 27 consecutive patients (mean age 58.1 ± 9.6 years and 22 female) with CSX 24 patients were found to have OSA according to PSG examination. CPAP therapy was applied to 17 patients (mean age 56.4 ± 8.6 years, 14 female) who accepted to participate in the treatment phase of the study. Following a three-month course of treatment, myocardial ischemia was reduced in 13 of the 17 patients. There were statistically significant correlations between the reduction in myocardial ischemia and patient’s diagnosis of hypertension (*p* = 0.006), higher serum HDL cholesterol levels (*p* = 0.009), and adherence to CPAP therapy (*p* = 0.047). **Conclusions:** The prevalence of OSA is significantly higher among the patients with CSX compared to the general adult population. In patients with CSX and OSA, improvement in myocardial ischemia was observed in MPS following CPAP therapy.

## 1. Introduction

Cardiac Syndrome X (CSX) is defined as evidence of myocardial ischemia such as ST segment depression during exertion, or impaired myocardial perfusion in myocardial perfusion scintigraphy (MPS) in a patient with anginal chest pain despite no obstructive coronary artery disease (CAD) on coronary angiography [1,2]. Generally, disturbances in the coronary microvascular circulation are implied for the pathophysiology of CSX. Consequently, the term “Microvascular Angina” (MVA) and this syndrome are considered to be synonymous. Despite the introduction of novel diagnostic criteria for CSX, the fundamental elements of these criteria remain as the presence of angina or angina-equivalent symptoms in patients with objective confirmation of myocardial ischemia through stress testing, and the absence of obstructive CAD [3]. The impact of CSX on mortality remains inconclusive, despite conflicting findings in the literature. However, there is a consensus that the syndrome is associated with an elevated risk of psychiatric morbidity [4,5,6,7]. The majority of patients with CSX are postmenopausal women [8]. Therefore, it has been suggested that estrogen deficiency may play a role in the pathogenesis of microvascular dysfunction [9]. Beyond traditional cardiovascular risk factors such as hypertension, smoking, diabetes, obesity and dyslipidemia, other non-traditional risk factors such as systemic inflammation, psychosocial stress, and OSA have emerged in recent years and gained attention for their potential role in CSX and other cardiovascular disorders. There is accumulating evidence highlighting a close relationship between inflammation and coronary microvascular dysfunction in various experimental and clinical settings. Chronic low-grade vascular inflammation plays important roles in the underlying mechanisms behind microvascular dysfunction [10]. Psychological stress is associated with adverse cardiovascular outcomes such as mental stress-induced myocardial ischemia. Psychological stress includes anxiety, depression, anger, and personality disturbances. Coronary microvascular dysfunction and coronary arterial spasm are phenotypes of coronary vasomotor disorders that are triggered by psychological distress and depression, thereby increasing cardiovascular disease risk [11]. 

**Figure 1:**
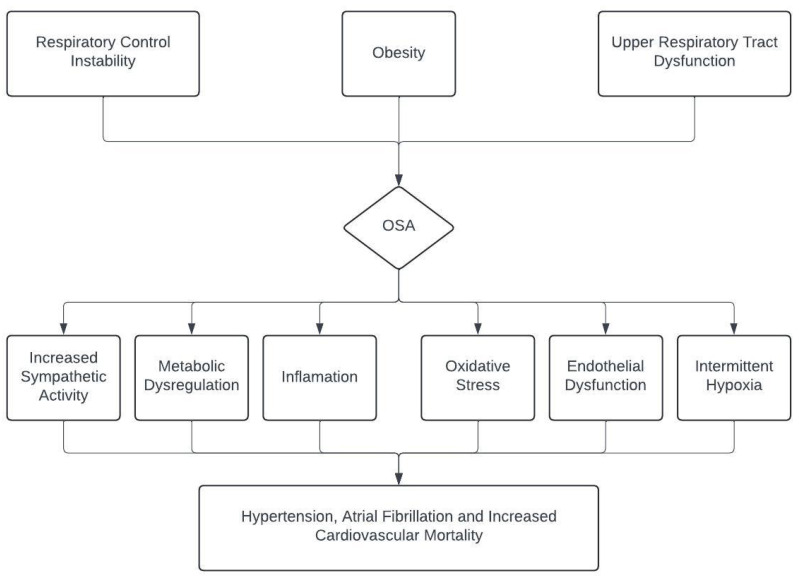
The cardiovascular impacts of OSA. OSA: obstructive sleep apnea.

Among these non-traditional conditions, OSA has emerged as a particularly important condition due to its strong association with cardiovascular morbidity. Obstructive sleep apnea (OSA) occurs when the upper respiratory tract closes for various reasons during sleep. Although OSA affects 20–30% of the general adult population, the prevalence of OSA increases to 40–80% of the patients with cardiovascular diseases including arterial hypertension, pulmonary hypertension, heart failure, CAD, atrial fibrillation, and ischemic stroke [12,13]. OSA and cardiovascular diseases share similar risk factors such as male gender, advanced age, and obesity. OSA has been demonstrated to exert acute and chronic adverse effects on the cardiovascular system (Figure 1). Increased sympathetic activity, metabolic dysregulation, inflammation, oxidative stress, endothelial dysfunction, and intermittent hypoxia caused by OSA result in poor cardiovascular outcomes. OSA also exerts a notable impact on preload and afterload which may affect cardiac functions [13]. OSA may also contribute to the development of CSX through the mechanism of microvascular circulatory disruption due to chronic hypoxia, inflammation, and oxidative stress [14,15]. Epidemiological studies on OSA have shown that the disease is particularly prevalent in men, in women with polycystic ovary syndrome, and in postmenopausal women, suggesting that estrogen may have a role in the pathophysiology of OSA. Clinical studies also indicate that estrogen replacement may provide benefits in OSA and its associated comorbidities [16]. While there are studies in the current literature investigating the association of OSA with CSX, there is a paucity of comprehensive studies examining the effects of OSA treatment on myocardial ischemia in patients with CSX [15,17].

The aim of this study was to estimate the prevalence of OSA in patients with CSX and to assess the impact of OSA treatment on anginal symptoms and myocardial ischemia.

## 2. Methods

This single-center prospective study was conducted at Marmara University Pendik Training and Research Hospital, Istanbul, Türkiye. The study was approved by the Marmara University Clinical Research Ethics Committee, and the study was conducted in accordance with the principles outlined in the Declaration of Helsinki. All patients gave written informed consent prior to participation in the study.

### 2.1. Study Population

The study population was selected consecutively from the patients who presented to the Cardiology Outpatient Clinic with angina or angina-equivalent complaints, were found to have ischemia on MPS, and were subsequently diagnosed with CSX via coronary angiography. In our study, CSX was defined as the objective demonstration of myocardial ischemia on a stress test (with MPS) performed in patients presenting with angina or angina-equivalent symptoms, along with findings of “normal coronary arteries” or “non-obstructive (<50%) CAD” on coronary angiography. Patients with previous myocardial infarction and previous percutaneous coronary intervention, MINOCA or coronary artery by-pass grafting surgery were excluded, since these conditions could not be regarded as CSX. To achieve standardization for MPS testing patients whose MPSs were performed at another center were also excluded.

### 2.2. Study Design

**Figure 2:**
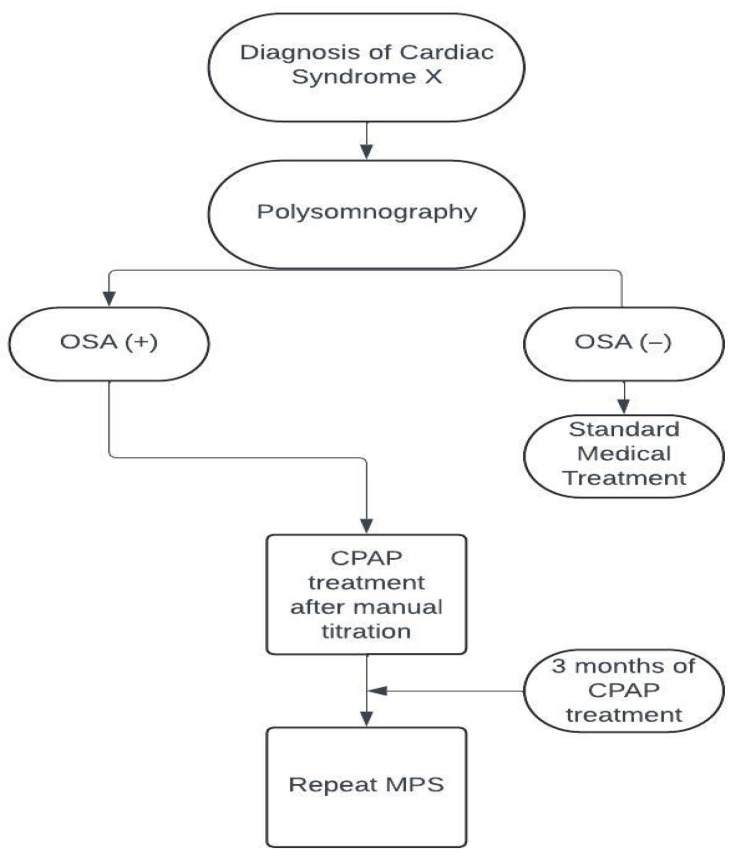
Study flowchart. CPAP: continuous positive airway pressure, MPS: myocardial perfusion scintigraphy, OSA: obstructive sleep apnea.

The study flowchart is shown in Figure 2. All patients underwent a complete cardiac evaluation. Demographic and clinical data of all patients were retrieved from the hospital database. Cardiovascular risk factors such as hypertension, diabetes, hyperlipidemia, and smoking were noted. Blood tests including hemoglobin and lipid panels were also noted. Canadian angina scores (CAS) of the patients were evaluated according to current guideline [18].

**Figure 3:**
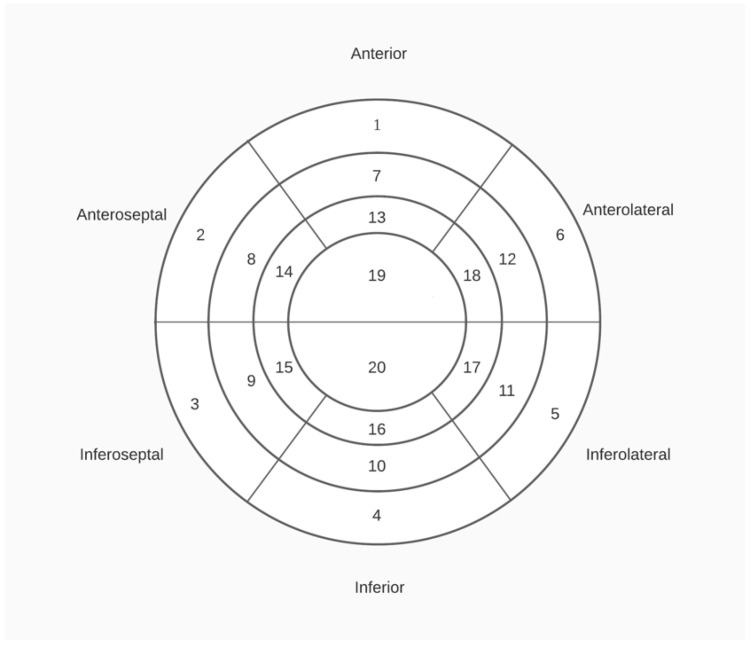
Twenty segment left ventricle model.

All patients underwent polysomnography (PSG) testing. The PSG was scored in accordance with the guidelines of the American Academy of Sleep Medicine (AASM) [19]. The test results were subsequently evaluated by a pulmonologist or a neurologist with a sleep certificate at our institution. Patients exhibiting an apnea–hypopnea index (AHI) of 15 or greater following the test were diagnosed with OSA. Continuous positive airway pressure (CPAP) treatment was initiated after calculating the optimal pressure values for each patient. Patients were interviewed at biweekly intervals during treatment, either in person or, if this was not feasible, via telephone. Patients were encouraged to utilize CPAP, and the mean duration of use was documented. Following a three-month course of nasal CPAP therapy, CASs of the patients were re-evaluated, and a control MPS was conducted to re-evaluate the ischemia findings. The MPSs of the patients were evaluated in accordance with the 20-segment model by the same nuclear medicine physician who also reassessed and compared the ischemic areas in the initial MPS examination. The 20-segment model, in which the ischemic segments of the left ventricle are evaluated in MPS, and the left ventricle is numbered starting from the basal segment and progressing towards the apical segment, is shown in Figure 3. To assess changes in symptoms and myocardial ischemia with greater objectivity, the patients did not receive any antianginal therapy, and no additional antianginal treatment was incorporated into the patients’ preexisting medical regimens during the study period.

### 2.3. Statistical Analysis

The IBM^®^ SPSS Statistics Version 26 program was employed for the purpose of statistical analysis. In the present study, the demographic and clinical characteristics of the patients were expressed as numbers and percentages for categorical variables and as mean and standard deviation (SD) for normally distributed continuous variables. In all analyses, a *p*-value of less than 0.05 was considered statistically significant. Categorical data were compared using chi-square or Fisher’s exact test, while Wilcoxon and two-sided Mann–Whitney U-test were used to compare continuous variables. Correlation analysis and logistic regression analysis were conducted to explore predictors of ischemia recovery.

## 3. Results

### 3.1. Baseline Characteristics

The study included 27 consecutive patients with CSX (median age 60.0 years and 22 female). Patient enrollment was conducted between September 2019 and September 2022. The characteristics of the patients are provided in Table 1. PSG revealed OSA in 24 patients (88.9%). All patients were given CPAP treatment. However, seven patients refused CPAP treatment and withdrew their consent. Therefore, the treatment phase of the study was continued with 17 patients (median age 57.0, 14 female).

### 3.2. Post-CPAP Findings

After 3 months of CPAP treatment, all 17 patients underwent control MPS, which revealed a decrease in myocardial ischemia in 13 patients. Table 2 shows the characteristics of the patients according to improvement of myocardial ischemia.

**Figure 4:**
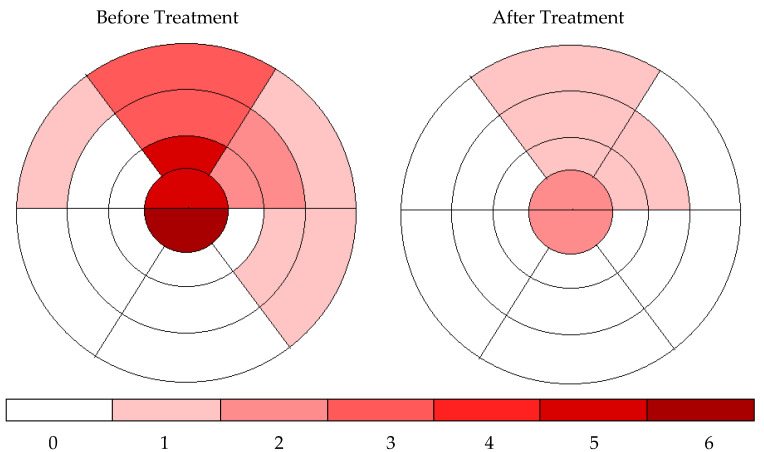
Total ischemic density of the study population before and after CPAP treatment (colors indicate the number of patients who had ischemia in that specific segment.).

The mean CAS decreased from a median of 2.0 to 1.0 with CPAP treatment in patients with decreased myocardial ischemia. However, the decrease was not statistically significant (*p* = 0.108). In the patients exhibiting no reduction in myocardial ischemia, there was no alteration in the mean CAS following CPAP treatment in comparison to the pretreatment assessment. The total number of ischemic segments and ischemic segment localization obtained by combining the ischemic segments detected in the MPS scans of the patients participating in the treatment phase of the study before and after CPAP treatment are shown in Figure 4.

Univariable and multivariable logistic regression analysis were conducted to assess the parameters associated with the presence of improvement in myocardial ischemia after three months of CPAP treatment (Table 3). In the univariable logistic regression analysis, improvement in myocardial ischemia was significantly correlated with the duration of CPAP device use, hypertension and HDL cholesterol. Multivariable logistic regression analysis revealed the duration of CPAP use as an independent predictor of improvement of myocardial ischemia.

## 4. Discussion

Since OSA and CSX share similar risk factors, we explored a possible association between OSA and CSX and found that nearly 90% of the patients with CSX were suffering from OSA. We further explored whether CPAP treatment might have a beneficial effect in the management of these patients and observed that CPAP treatment improved myocardial ischemia in 76% of the patients.

Despite the numerous hypotheses regarding the underlying cause of myocardial ischemia and the management of ischemic symptoms in patients with CSX, the precise etiology and most effective treatment remain uncertain. Intermittent hypoxia and vascular inflammation play key functions in the pathophysiology of CSX. Hypoxia activates transcription factor nuclear factor KB and hypoxia-inducible factor-1, which increases the expression of genes encoding proteins such as erythropoietin, vascular endothelial growth factor, and inducible nitric oxide synthase [20]. Therefore, there may be a strong relation between CSX and OSA, as OSA is also associated with hypoxia and increased vascular inflammation. Previous studies exploring the relation of OSA and CSX have reported prevalence of OSA ranging from 61.1% to 87% [17,21,22]. Similarly, the prevalence of OSA was 88.9% in our cohort.

No universal treatment modality has been established for CSX due to the syndrome’s heterogeneous nature. This situation compels researchers to assess novel avenues in etiology and treatment. Although there are data showing the relation between CSX and OSA, no study has yet clearly demonstrated the effects of CPAP treatment on myocardial ischemia in CSX patients. The background of OSA is characterized by intermittent hypoxia, which may result in endothelial dysfunction, increased sympathetic activity, elevated oxidative stress, metabolic dysregulation, and chronic inflammation, which may all eventually cause microvascular dysfunction [13,23]. Hypoxia in OSA patients may increase blood pressure, and induce vascular smooth-muscle remodeling and cardiac hypertrophy, which leads to an increase in the oxygen consumption of the heart aggravating myocardial ischemia. Therefore, improvement of hypoxia might result in improvement on the endothelial functions, oxidative stress and inflammatory status. Following a three-month course of CPAP therapy, we found that myocardial ischemia improved in 13 CSX patients (76.4%). The CASs of the patients also decreased by CPAP therapy, although the difference did not reach statistical significance, which may be explained by the small sample size of our study. Our results suggest the use of CPAP therapy as an additional therapy in CSX patients, especially if they also have OSA symptoms. We found that CPAP therapy was more effective in reducing myocardial ischemia in hypertensive patients compared to non-hypertensive patients. OSA is a well-known risk factor for hypertension, and CPAP therapy might be more effective in resolving myocardial ischemia, as it also helps to control blood pressure.

In our cohort, the majority of patients were postmenopausal women. This sex-specific pattern has been attributed to differences in coronary microvascular function, hormonal influences such as estrogen deficiency, and a higher prevalence of comorbid conditions including hypertension, metabolic syndrome, and autoimmune diseases in women. Notably, symptomatic women with non-obstructive CAD have been shown to have a significantly higher risk of both fatal and non-fatal cardiovascular events within five years [24]. Several studies have demonstrated that women with cardiovascular disease often receive less aggressive diagnostic evaluation and therapeutic interventions, which may contribute to worse long-term outcomes [25,26]. These disparities highlight the importance of taking gender-specific factors into account when diagnosing, risk-stratifying, and managing CSX, especially when there are other conditions present, such as OSA, which can worsen microvascular dysfunction and increase the risk of adverse cardiovascular events.

HDL, the principal mediator of reverse cholesterol transport, possesses not only anti-atherosclerotic but also anti-inflammatory and antioxidant properties [27]. In a study investigating lipid profiles in patients with CSX, low serum HDL levels were found to be associated with systemic inflammation [28]. In our study, patients who demonstrated a reduction in myocardial ischemia following three months of CPAP therapy had significantly higher serum HDL levels compared to those in whom ischemia persisted (*p* = 0.006). These findings suggest that higher baseline serum HDL levels in patients with CSX may contribute to the improvement of myocardial ischemia with CPAP therapy, potentially by attenuating systemic inflammation and oxidative stress. Improvement in myocardial ischemia was not seen in four of the patients, who had median age and AHI values were higher and shorter CPAP use duration, although the differences were not statistically significant. In their study, Goyal et al. also identified older age, higher AHI, higher BMI, and lower oxygen saturation as predictors of CPAP treatment failure [29]. Therefore, non-adherence to CPAP treatment might explain the persisting myocardial ischemia in these patients. However, in order to maintain CPAP therapy compliance, the patients were encouraged on a biweekly basis through face-to-face interviews or telephone calls.

In light of the above-mentioned findings, the clinical take-home message from our research is that OSA may accompany CSX patients, and the treatment of OSA by CPAP therapy may improve the myocardial ischemia in these patients, especially if they also have hypertension. Therefore, patients with CSX should be carefully evaluated for OSA symptoms and the presence of OSA should be further confirmed by PSG if symptoms are present. CPAP treatment for OSA may also help control myocardial ischemia and angina.

### 4.1. Strengths

Our study is the first to investigate the effects of OSA treatment on myocardial ischemia in CSX patients. Our research provides a different perspective on the treatment of CSX and shows the positive effects of CPAP treatment on myocardial ischemia. Myocardial ischemia was not only assessed by subjective angina scores but also demonstrated by objective MPS testing.

### 4.2. Study Limitations

Our study has certain limitations. The small sample size and the fact that it is a single-center study are the main limitations of the study. The lack of a power analysis to estimate the sample size in the study is another limitation. CFR measurement is among the criteria recommended by the COVADIS group for the definitive diagnosis of MVA [30]. However, CFR was not routinely measured in our clinic. Therefore, the lack of proving MVA by CFR is another limitation of our study. We did not assess inflammatory markers, which may help to elucidate the underlying mechanisms in the improvement of myocardial ischemia. CPAP treatment was administered for only three months, and the patients were encouraged to receive CPAP treatment adequately. However, there might be nonadherence to CPAP treatment, and our results cannot provide conclusion about its long term efficacy.

## 5. Conclusions

OSA was highly prevalent in patients with CSX. CPAP treatment improved myocardial ischemia in most of the patients. OSA may be regarded as a modifiable risk factor for CSX. Our results suggest that all patients diagnosed with CSX should be evaluated for OSA symptoms and undergo PSG if necessary, as treatment of OSA by CPAP may help angina symptoms and myocardial ischemia in these patients. Further large-scale studies are required to investigate the relation between CSX and OSA, and the long-term efficacy of CPAP therapy in patients with CSX and concomitant OSA.

## Figures and Tables

**Table 1:** Baseline characteristics of the patients.

	*n* = 27
Age [years, median (IQR)]	60.0 (50.5–64.0)
Female sex (*n*—%)	22 (81.5)
BMI [kg/m^2^, median (IQR)]	33.2 (29.54–36.4)
Hypertension (*n*—%)	22 (81.5)
Diabetes Mellitus (*n*—%)	11 (40.7)
Dyslipidemia (*n*—%)	8 (29.6)
Hypothyroidism (*n*—%)	5 (18.5)
Smokers (*n*—%)	9 (33.3)
Canadian Angina Score [median (IQR)]	2.0 (1.0–2.0)
Obesity (*n*—%)	19 (70.4)
OSA (*n*—%)	24 (88.9)
Hemoglobin [g/dL, median (IQR)]	13.6 (12.7–14.15)
Triglyceride [mg/dL, median (IQR)]	156.0 (116.15–199.0)
LDL cholesterol [mg/dL, median (IQR)]	115.0 (98.0–144.0)
HDL cholesterol [mg/dL, median (IQR)]	46.0 (39.8–51.05)

BMI: body mass index, HDL: high-density lipoproteins, LDL: low-density lipoproteins, OSA: obstructive sleep apnea.

**Table 2:** Characteristics of the patients according to post-CPAP MPS ischemia results.

	Reduced Ischemia (*n* = 13)	Persisting Ischemia (*n* = 4)	*p*
Age [years, median (IQR)]	57.0 (49.5–63.0)	58.5 (48.75–66.75)	0.703 ^a^
Female sex (*n*—%)	11 (84.6)	3 (75.0)	1.00 ^b^
BMI [kg/m^2^, median (IQR)]	34.1 (30.14–37.55)	33.0 (28.8–46.65)	0.785 ^a^
Hypertension (*n*—%)	13 (100)	1 (25)	0.006 ^b^
Diabetes Mellitus (*n*—%)	6 (46.2)	1 (25)	0.603 ^b^
Dyslipidemia (*n*—%)	5 (38.5)	0	0.261 ^b^
Hypothyroidism (*n*—%)	3 (23.1)	1 (25)	1.00 ^b^
Smokers (*n*—%)	4 (30.8)	2 (50)	0.584 ^b^
Hemoglobin [g/dL, median (IQR)]	13.4 (12.35–14.4)	14.1 (13.62–15.25)	0.296 ^a^
WBC [×10^3^/µL, median (IQR)]	7.1 (6.3–9.45)	7.7 (6.6–9.3)	1.0 ^a^
Triglyceride [mg/dL, median (IQR)]	191 (134.5–242.0)	135.5 (111.3–176.8)	0.163 ^a^
HDL cholesterol [mg/dL, median (IQR)]	50.6 (45.4–54.35)	39.8 (34.42–41.72)	0.006 ^a^
LDL cholesterol [mg/dL, median (IQR)]	121 (107.0–144.0)	128.5 (88.25–169.5)	0.956 ^a^
AHI [median (IQR)]	27.4 (20.05–42.4)	28.1 (15.5–63.07)	1.0 ^a^
REM AHI [median (IQR)]	55.15 (26.52–70.2)	54.05 (40.95–65.95)	1.0 ^a^
ESS [median (IQR)]	8.0 (2.5–13.5)	9.5 (5.25–10.0)	0.703 ^a^
Duration of CPAP use [hours/day, median (IQR)]	7.0 (5.0–8.0)	5.0 (4.25–5.75)	0.060 ^a^

^a^ Mann–Whitney U test, ^b^ Chi-squared. AHI: Apnea–hypopnea index, BMI: body mass index, CPAP: continuous positive airway pressure, ESS: Epworth Sleepiness Scale, HDL: high-density lipoproteins, LDL: low-density lipoproteins, MPS: myocardial perfusion scintigraphy, REM: rapid eye movement.

**Table 3:** Parameters associated with improvement in myocardial ischemia after 3 months of CPAP therapy.

	Odds Ratio	%95 Confidence Interval	*p* Value
Univariable			
Age	0.97	0.83–1.12	0.969
Male sex	0.55	0.04–8.27	0.662
BMI	0.94	0.78–113	0.508
Hypertension	14	2.11–92.54	0.006
Diabetes Mellitus	2.57	0.21–31.71	0.461
Hypothyroidism	0.90	0.07–12.18	0.937
Obesity	3.33	0.32–34.83	0.315
Smoking	0.44	0.05–4.37	0.487
LVEF	1.33	0.95–1.85	0.096
HDL	1.818	1.928–3.562	0.009
AHI	1.00	0.95–1.05	0.918
REM AHI	0.99	0.94–1.04	0.992
ESS	0.98	0.77–1.26	0.989
Duration of CPAP use (hours/day)	5.60	1.03–30.62	0.047
Multivariable			
Age	0.95	0.77–1.17	0.621
BMI	0.99	0.74–1.32	0.926
Duration of CPAP use (hours/day)	5.91	0.99–35.27	0.049

AHI: Apnea–hypopnea index, BMI: body mass index, CPAP: continuous positive airway pressure, ESS: Epworth Sleepiness Scale, HDL: high-density lipoproteins, LVEF: left ventricular ejection fraction, REM: rapid eye movement.

## Data Availability

The data that support the findings of this study are available from the corresponding author upon reasonable request.

## Abbreviations

CSX	Cardiac Syndrome X
OSA	Obstructive Sleep Apnea
CPAP	Continuous Positive Airway Pressure
MPS	Myocardial Perfusion Scintigraphy
CAD	Coronary Artery Disease
MVA	Microvascular Angina
PSG	Polysomnography
AHI	Apnea–Hypopnea Index
CAS	Canadian Angina Score
HDL	High-Density Lipoprotein
LDL	Low-Density Lipoprotein
BMI	Body Mass Index
ESS	Epworth Sleepiness Scale
LVEF	Left Ventricular Ejection Fraction
REM	Rapid Eye Movement (sleep phase)
ACE-I	Angiotensin-Converting Enzyme Inhibitor
ARB	Angiotensin Receptor Blocker
OAD	Oral Anti-Diabetic
CFR	Coronary Flow Reserve
